# Observations on the Mineral Waters of Moffat, in the County of Dumfries, Scotland

**Published:** 1799-11

**Authors:** T. Garnett

**Affiliations:** *Professor of Physics and Philosophy in* Anderson's University, Glasgow, &c.


### *Obſervations on the Mineral Waters of* Moffat, *in the County of* Dumfries, Scotland, *by* T. Garnett, *M. D. Profeſſor of Phyſics and Philoſophy in* Anderson's Univerſity, Glaſgow, *&c.*

THE village of Moffat is ſituated on a riſing ground, at the head of a plain, or valley, extending more than twenty miles along the banks of the Annan. It is encompaſſed on the eaſt, north, and weſt, by hills of different heights. It conſiſts chiefly of one very ſpacious ſtreet, with two good inns, and ſeveral private lodging houſes, which are let to invalids, who reſort to this place during the ſummer, for the ſake of its mineral waters. The church is a handſome building, ſurrounded by trees, which produce a good effect. Indeed, the view of the village from any part is far from unpictureſque. The number of inhabitants is ſomething more than a thouſand. Lord Hopeton has a houſe here, at which he occaſionally reſides.

Moffat has long been celebrated for its mineral waters, and may be denominated the Harrogate of Scotland; numbers of invalids from Edinburgh, Glaſgow, Dumfries, and various parts of Scotland, reſort to it every year; and though in winter a reſidence here would be very dull and dreary, in ſummer the village is all life.

The climate of Moffat is ſaid to be remarkably healthy, and the air ſo extremely pure, as to occaſion ſneezing, and other marks of ſuperoxygenation, in perſons not accuſtomed to it, particularly if they have lived for ſome time in a large town, or a confined ſituation. Its effects are particularly exhilarating and bracing, as I have myſelf experienced; and though the ſhowers of rain are frequent, and ſometimes heavy, as might be expected in a mountainous country, yet a moiſt or foggy atmoſphere is ſeldom ſeen. Every opening of the clouds diſcovers a ſky of a beautiful azure, which, in a clear day, aſſumes a diſtinctneſs and brightneſs that might vie with an Italian ſky. Theſe circumſtances, with exerciſe, contribute, perhaps, more than the waters, to reſtore the exhauſted and debilitated conſtitution.

The mineral waters are of two kinds, ſulphureous and chalybeate: The firſt has long been diſtinguiſhed by the name of the Moffat well; and is ſituated about a mile and a half from the village. A good carriage road has been made to it, and there is a room and ſtables, near the the well, for the accommodation of the company while drinking the waters.

The ſpring oozes out of a rock, at the diſtance of only two or three yards from a little rivulet; a few yards above it is a bog, from whence it probably derives its ſulphureous impregnation. The well is covered over with a ſtone building, incloſing a pump. The water has a ſtrong ſmell, reſembling bilge water, or the ſcourings of a foul gun, like the ſulphureous waters at Harrogate, though not quite ſo ſtrong; it has a ſlight ſaline taſte, and ſparkles conſiderably when firſt taken from the ſpring, particularly when poured out of one glaſs into another. The ſides of the well are lined with a whitiſh cruſt; and when the water has been ſuffered to ſtand for ſome days without pumping, it becomes covered with a white film; both theſe, when dried, burn with a bluiſh flame and ſuffocating ſmell, which indicate them to be ſulphur.

On the 9th of October, when the temperature of the air was 54°, and that of the adjoining brook 48°, the temperature of the ſpring was 50°. The following experiments were made with a view of aſcertaining the nature of its contents:

1. Characters written upon paper with *acetite of lead*, were rendered viſible on being immerſed in the water; the colour was at firſt brown, and on remaining longer quite black.

2. A ſolution of acetite of lead in diſtilled water, dropped into the water, cauſed a copious brown precipitate.

3. Tincture of galls produced no change.

4. Lime-water produced a very ſlight turbidneſs.

5. Tincture of turnſole produced ſcarcely any ſenſible redneſs.

6. Acid of ſugar produced no change.

7. Muriat of barytes produced no effect.

8. Nitrat of ſilver cauſed a white cloudy appearance, with a copious precipitate.

9. When the water had been boiled for a few minutes, it was not changed by any of theſe precipitants, except the nitrat of ſilver.

From the firſt and ſecond of theſe experiments, it appears that the water is impregnated with ſulphurated hydrogen gas; the third ſhows it to contain no iron; the fourth and fifth indicate but a ſmall quantity of carbonic acid; from the ſixth it appears to contain no lime; and from the ſeventh no ſulphuric acid; the eighth, however, diſcovers the muriatic acid, which we ſhall afterwards find is combined with ſoda.

10. By

10. By means of the pneumatic apparatus, which I defcribed in a treatife I publifhed fome years ago on the Crefcent water at Harrogate, and likewife in an early number, I think the fecond or third of the New London Medical Journal, printed for Deighton, Holborn, nineteen cubic inches of permanently elaftic fluid were procured from a wine gallon of the Moffat water; of which, four were azotic gas; five carbonic acid, and ten fulphurated hydrogen gas,

11. A wine gallon of this water was evaporated very flowly to drynefs, and 36 grains of muriat of foda were obtained, fome of the cryftals of which were very diftinct.

Hence it may be concluded, that a wine gallon of the fulphur water at Moffat contains

| | | |
|---|---|---|
| Of muriat of foda — | 36 | grains. |
| Sulphurated hydrogen gas | 10 | cubic inches. |
| Azotic gas — — | 4 | |
| Carbonic acid gas — | 5 | |

This water will not keep; for though clofely corked up in bottles, in the courfe of two or three days it is found to have loft the whole of its fulphureous fmell.

The next water which I examined was the Hartfell Spa, which fprings out of the bafe of a mountain of that name, and is nearly five miles diftant from Moffat. It is found at the bottom of a deep and narrow ravine, or linn, the fides of which are entirely laid bare to the very top, and form a very interefting object to the mineralogift, as all the different ftrata can be diftinctly feen. Thefe ftrata dip towards the bottom of the mountain, and are inclined to the horizon in an angle of about 15°.

The loweft ftratum is a black and foft kind of rock which eafily crumbles to pieces, and confifts of clay, with great quantities of fulphuret of iron and fulphuret of alumin; immediately above this ftratum, which is feveral feet in thicknefs, lies another, compofed chiefly of clay and argilaceous iron-ftone; above this is another ftratum of blackifh fhale, refembling the loweft; and above this another of argilaceous iron-ftone, of a fine deep red. The afcent up this ravine is very difficult;—a little brook tumbles down it, forming fome pretty cafcades, and nearly at the foot of the glen is the mineral water, which feems to originate from water filtering through and diffolving the fulphats of iron and alumin of the rock; and in confequence of this it is, as might be expected, ftrongeft after rains, contrary to moft mineral waters.

waters. The whole brook depofits an ochre, or oxid of iron, which colours the rocky channel to a confiderable diftance. On fome of the rocks above the fpring I found fome beautiful fpecimens of *alumen plumofum*, and a few green cryftals of fulphats of iron.

In thefe fchiftous ftrata, the fulphurets are decompofed; by the action of the air, and the contact of water, the fulphur is converted into fulphuric acid, which combining with the iron and alumin, form the fulphats; thefe being foluble in water, are wafhed away, filter along the crevices, and iffue in the form of a fpring, which is covered by a fmall building.

This well was difcovered in the year 1748, by JOHN WILLIAMSON, an excentric but benevolent character. He believed in the Pythagorean doctrine of the *metempfychofis*, or tranfmigration of the foul into the bodies of different animals; on this account he never tafted animal food for the laft forty or fifty years of his life, nor would he fuffer the fmalleft infect to be killed if he could prevent it. He drank this water conftantly for many years.

The water is perfectly clear when taken from the well, but gradually depofits, even though fealed up, a little oxid of iron in the form of a fine fediment. It has a ftrong aftringent tafte like ink. The following is the refult of the experiments which I made upon it:

1. Tincture of galls dropped into it produced a colour nearly as black as ink, and this colour was as deep when the experiment was made after the water had been boiled as it was before, which fhows that the iron is not fufpended by the carbonic but by a fixed acid.

2. Muriat of barytes produced a white coloured and a copious fediment.

3. Acid of fugar produced no change.

4. Acetite of lead produced a flight turbidnefs with a white precipitate.

5. Tincture of turnfole was rendered a little red.

6. Lime water produced a flight turbidnefs, with fome precipitate of alumin.

7. By means of the machine, only five cubic inches of gas were expelled from a wine gallon of the water, which was chiefly azotic gas.

8. A wine gallon of the Hartfell water was made to boil gently; it foon became turbid, and depofited a brown powder, after which it was perfectly clear. The powder was collected by filtration, and found to weigh weigh fifteen grains; it was of a yellowifh colour, but changed to a beautiful red on expofure to a confiderable heat: it was found to be oxid of iron.

The clear liquor was evaporated very gently to drynefs, and the faline matter procured in this manner weighed 96 grains. This was found to confift of fulphat of iron, and fulphat of alumin. In order to difcover the refpective quantities of each of thefe falts, the whole was diffolved in water, and the iron precipitated by tincture of galls. When this was feparated, a folution of falt of tartar (carbonat of pot-afh) was added, which precipitated the alumin in a carbonated ftate, and from the quantity of carbonat of alumin, it was eafy to calculate the fulphat of alumin, which I found to be 12 grains; the quantity of fulphat of iron muft therefore be 84 grains.

This water taftes much ftronger after it has ftood two or three days, even in an open veffel, though it is in fact weaker, becaufe it has loft part of its iron by ftanding. The fulphuric acid lofing part of its iron, its tafte becomes more fenfible, and the water approaches nearer to a folution of fal martis.

From the preceding experiments, it appears that a wine gallon of the Hartfell water contains,

| | | |
|---|---|---|
| Of fulphat of iron, | — — | 84 grains. |
| Sulphat of alumin, | — — | 12 grains. |
| Azotic gas, | — — — | 5 cubic inches. |

Together with fifteen grains of oxid of iron, with which the fulphuric acid feems to be fuperfaturated, and which it gradually depofits on expofure to the air, and almoft immediately if boiled.

As the principal mineralifers of this water are the fulphats of iron and alumin, it is evident, that if well corked, it will keep for months, and perhaps years, unimpaired in its qualities: hence it may be carried to a diftance, better than moft mineral waters, and its good effects need not be confined to Scotland, or even to Britain. When Dr. JOHNSTONE, a phyfician at Moffat, had the care of it, he fent it to many towns in England, and to the Weft Indies; but it is now in hands that render it of comparatively little benefit to the public. As this water keeps fo well, it is not neceffary to go to the fpot to drink it, but it may be procured in Moffat in a very frefh ftate. It very much refembles the water of the Horley-Green Spa, near Halifax; of which I publifhed publiſhed an analyſis in 1790; only the Horley-Green water is conſiderably ſtronger.

While rambling about Moffat, I one day obſerved a ſpring near the Evan Bridge, at the end of the town, beyond the Manſe on the Dumfries road, which appeared to be chalybeate. On taſting it, I found it ſtrongly reſembled the chalybeates at High Harrogate; I therefore made ſome experiments with it, of which the following are the reſults:

1. Tincture of galls produced a beautiful purple colour, but not after the water had been boiled.

2. Lime water produced a ſlight cloud.

3. Muriat of barytes cauſed no change.

4. Acid of ſugar produced no effect.

5. Tincture of turnſole cauſed a ſlight redneſs.

6. Acetite of lead produced no effect.

Theſe experiments convinced me of its reſemblance to the Harrogate chalybeates, in which the iron is ſuſpended by carbonic acid, as is evidently the caſe here.

I next expelled the gas by means of the machine, which I found to amount to ſeventeen cubic inches, of which thirteen were carbonic acid, and three azotic gas.

A wine gallon of the water was next made to boil gently for a quarter of an hour, during which time it depoſited a quantity of yellow ſediment, which being collected by filtration, weighed two grains, and was evidently oxid of iron. The clear liquor which remained after filtration was not affected by any of the above teſts.

Hence a wine gallon of this water muſt contain,

| | |
|---|---|
| Of carbonat of iron, — — | 2 grains, |
| Carbonic acid gas, — — — | 13 cubic inches. |
| Azotic gas, — — — | 3 ditto. |

The quantities of iron and carbonic acid gas, which are the only ſubſtances of any conſequence, are very nearly equal to thoſe in the chalybeates at Harrogate. (See Memoirs of the Medical Society of London, Vol. V. and my Treatiſe on the Mineral Waters of Harrogate.) From this it cannot be doubted, that if this well were properly encloſed, which I was promiſed ſhould be done, it would be a valuable addition to Moffat. It would agree with many conſtitutions, in which the Hartfell water is improper, on account of its too great aſtringency and tonic power; and its vicinity to Moffat is a great advantage, as it can be drank on the ſpot by thoſe who reſort to this watering place.

